# Usefulness and limitations of transthoracic echocardiography in heart transplantation recipients

**DOI:** 10.1186/1476-7120-6-2

**Published:** 2008-01-11

**Authors:** Sergio Mondillo, Massimo Maccherini, Maurizio Galderisi

**Affiliations:** 1 Cardiologia Universitaria, Università di Siena, Siena, Italy; 2 UO Complessa Cardiochirurgia dei Trapianti, AUO di Siena, Siena, Italy; 3 Cardioangiologia con UTIC, Dipartimento di Medicina Clinica e Sperimentale, Università Federico II, Napoli, Italy

## Abstract

Transthoracic echocardiography is a primary non-invasive modality for investigation of heart transplant recipients. It is a versatile tool which provides comprehensive information about cardiac structure and function. Echocardiographic examinations can be easily performed at the bedside and serially repeated without any patient's discomfort. This review highlights the usefulness of Doppler echocardiography in the assessment of left ventricular and right ventricular systolic and diastolic function, of left ventricular mass, valvular heart disease, pulmonary arterial hypertension and pericardial effusion in heart transplant recipients. The main experiences performed by either standard Doppler echocardiography and new high-tech ultrasound technologies are summarised, pointing out advantages and limitations of the described techniques in diagnosing acute allograft rejection and cardiac graft vasculopathy. Despite the sustained efforts of echocardiographic technique in predicting the biopsy state, endocardial myocardial biopsies are still regarded as the gold standard for detection of acute allograft rejection. Conversely, stress echocardiography is able to identify accurately cardiac graft vasculopathy and has a recognised prognostic in this clinical setting. A normal stress-echo justifies postponement of invasive studies. Another use of transthoracic echocardiography is the monitorisation and the visualisation of the catheter during the performance of endomyocardial biopsy. Bedside stress echocardiography is even useful to select appropriately heart donors with brain death. The ultrasound monitoring is simple and effective for monitoring a safe performance of biopsy procedures.

## Background

Over the past decade heart transplantation (HT) has evolved from a rarely performed procedure to an accepted therapy for advanced heart failure. About 45% of the candidates to HT have ischemic cardiomyopathy while 55% have some form of dilated cardiomyopathy of various origin. The prognosis for HT patients following the orthotopic procedure has greatly improved over the past 20 years, and a recent report (August 2006) of Heart Transplants: Statistics of American Heart Association. informs that the 5 years survival rates is 66.9% in women and 71.2% in men. Although significant advances have been reached in surgical techniques, in donor and recipient selection criteria, and also in the management of transplant patients, allograft rejection remains the most important cause of morbidity and. the primary limitation for the survival of these patients.

Acute allograft rejection (AAR) is frequent in the first months after HT. Because it is initially asymptomatic, regular rejection surveillance is obligatory by monitoring immunosuppressive treatment, clinical and laboratory data and, in particular, by performing endomyocardial biopsies (EMBs), which represent the gold standard for the detection of rejection. AAR is characterised histologically by inflammatory cell infiltrates, interstitial edema and myocite necrosis which ultimately translates into structural and functional abnormalities of the allograft. A first international grading system for cardiac allograft biopsies, adopted in 1990 by the International Society for Heart Transplantation [1], has been updated in 2004 by the International Society for Heart and Lung Transplantation (ISHLT) (Table 1) [2].

**Table 1:** Grading of rejection of endomyocardial biopsy according to International Society of Heart and Lung Transplantation (update 2004)

**Grade**	**Signs of rejection**
Grade 0 R	No acute cellular rejection
Grade 1 R	Mild, low-grade, acute cellular rejection
Grade 2 R	Moderate, intermediate-grade, acute cellular rejection
Grade 3 R	Severe, high-grade, acute cellular rejection

Long-term survival of allografted hearts is limited by a progressive fibro-proliferative disease, resulting in intimal thickening and occlusion of the grafted coronary vessels. This disease, variously defined as accelerated transplant coronary artery disease (CAD) or cardiac graft vasculopathy (CAV), is also known as chronic allograft rejection [3]). After the first year transplant peripheral and cerebro-vascular disease, end-stage renal disease, malignancy and infections are the other main causes of death. Specific side-effects of the immunosuppressive agents like renal failure, arterial hypertension and diabetes mellitus shall be also taken into account.

### The role of Doppler echocardiography in the transplanted heart

Echocardiography is particularly useful for the assessment of HT recipients since it is easily performable and not associated with the risks of the invasive procedures. Its versatility allows it to be applied in a wide variety of situations in the post-transplant setting.

#### The echocardiographic features of left and right atria

**Figure 1:**
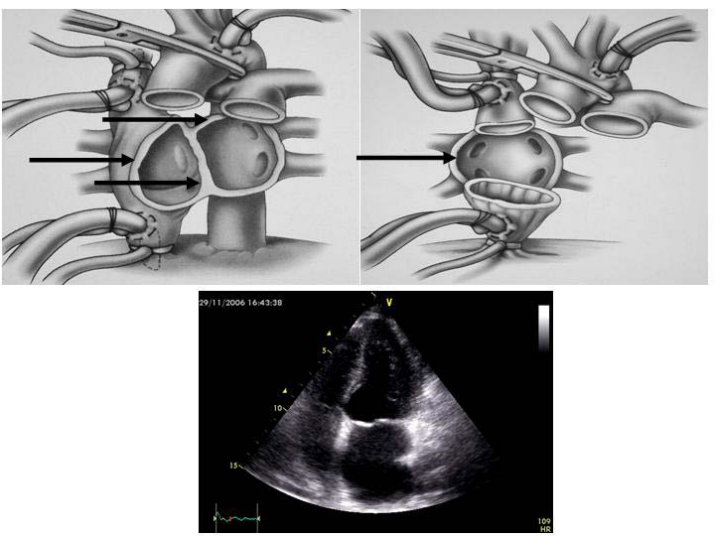
In the upper left panel a pericardial view (frontal) showing the edge of both atria which are preserved for conventional surgical technique (*Shumway technique*): possibility of interatrial ridge are dependent of the redundancy of donor as well as recipient residual tissue (arrows). In the upper right panel pericardial view (frontal) showing how the edge of both atria are removed for *bicaval surgical technique*: possibility of interatrial ridge are dependent of the redundancy of recipient septal residual tissue only (arrow). In the lower panel 2-D echocardiographic apical 4-chamber view showing left atrial enlargement and point of suture of a transplanted heart. Mod from Heart Transplantation – Churcill Livingstone – James K. Kirklin et al. 2002.

During HT procedure donor cardiectomy involves only partial atrial resection. With the standard surgical procedure (*biatrial surgical approach*) an anastomosis is made between the residual recipient atrial tissue and the donor atria such that there is a characteristic unique echocardiographic atrial morphological appearance. Therefore, transplanted hearts have increased size in both atria, primarily caused by an increase in their long-axis dimension. Sometimes, an echo dense ridge is also visualisable at mid-atrial level (best appreciated in apical 4-chamber view), it being the site of anastomosis between the residual recipient atrial tissue and the donor atria [4] (Figure 1). The standard surgical technique has been reported to contribute to sub-optimal hemodynamics, specifically abnormal patterns of LV filling and to predispose to atrial thrombus formation, due to blood stasis in the dilated atrial cavities [5]. It is not unexpected, therefore, that post-HT echocardiographically determined left atrial area is inversely correlated with patient's survival [4]. The *bicaval operative technique*, introduced in the last decade, is associated with better preservation of atrial morphology [6] and lower risk for development of atrial thrombus [7].

#### Assessment of left ventricular systolic function

The normalisation of left ventricular (LV) systolic function (M-mode derived endocardial fractional shortening or 2-D ejection fraction [EF])) after successful HT is responsible for early improvement of symptoms and has a strong impact on prognosis. In AAR LV systolic function is rarely affected but an early alteration of fractional shortening predicts subsequent development of CAV [8]. On a cohort of 65 HT recipients, 10–15 years after surgery, LV chamber dimension were well maintained and mean EF was 63% [9]. LV systolic dysfunction late after HT is often due to the effects of CAV and is associated to a poor prognosis [10]. Worthy of note, pulsed Tissue Doppler derived myocardial systolic velocity (S_m_) of ≤ 10 cm/s has been found to be associated with a 97.2% likelihood for transplant CAD whereas S_m _values of > 11 cm/s exclude CAD with 90.2% probability [11].

### Assessment of left ventricular diastolic function

**Figure 2:**
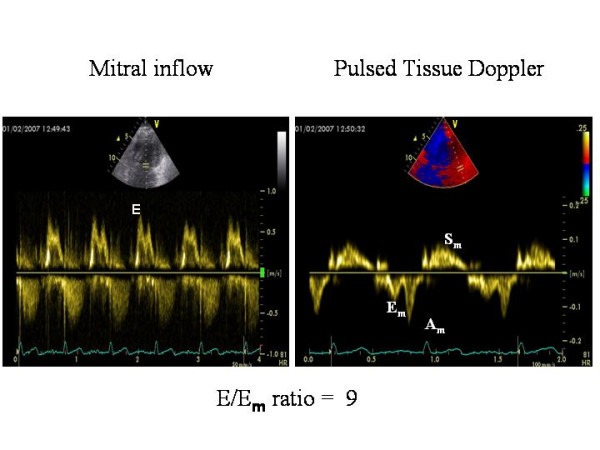
Standard Doppler derived transmitral inflow pattern (left panel) and Pulsed Tissue Doppler of the lateral mitral annulus in a HT recipient. The ratio is = .9.

**Figure 3:**
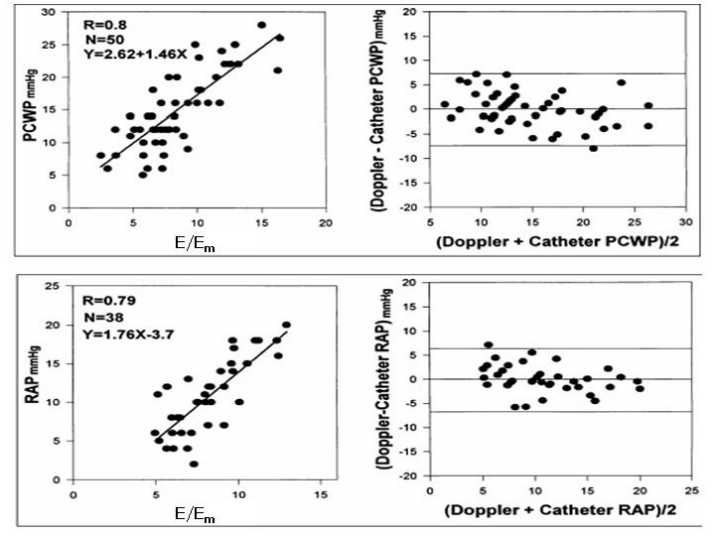
In the upper panel relation (left) and plot of Bland-Altman (right) between pulmonary capillary wedge pressure (PCWP) and mean mitral E/Ea ratio in transplant recipients. In the lower panel relation (left) and plot of Bland-Altman (right) between invasive mean right atrial pressure (RAP) and mean tricuspid E/Ea ratio in transplant recipients. (mod from Sudereswaran et al, Am J Cardiol 1998).

Although early AAR can be often responsible for the development of LV restrictive pattern, the recent literature does not support the use of Doppler indices of LV diastolic function as markers of AAR [12]. Standard Doppler appears as a method with an excellent specificity but insufficient sensitivity and this is mainly due to the influence of recipient atrial contraction timing on early ventricular filling and to sinus tachycardia which induces a frequent overlapping between E and A velocities [13]. Preserving left atrial morphology by using surgical bicaval techniques permits the maintenance of normal ventricular filling dynamics [4]. Because of the relatively load independency of myocardial velocities demonstrated also in HT recipients [14], pulsed Tissue Doppler has been proposed for the evaluation of LV diastolic properties due to AAR [11,15,16]. In the experience of Dandel et al, the negative and positive predictive values for AAR and transplant CAD of Tissue Doppler derived myocardial early diastolic velocity (E_m_) appeared high enough (= 92–96%) to allow a reliable non-invasive monitoring, instead of routinely scheduled EMBs [11]. Another study, which used A_m _velocity, has reported less favourable data, with good sensitivity (= 82%) but low specificity (= 53%) in predicting significant AAR [17]. In 50 HT patients, assessed by either right-sided cardiac catheterisation and Doppler echocardiography (including pulsed Tissue Doppler of the mitral annulus) simultaneously, mean wedge pressure was related weakly to mitral inflow variables but strongly to E/E_m _[r = 0.80; wedge pressure = 2.6+1.46(E/E_m_)] [18] (Figure 3), an index validated also in the general clinical setting (19). Figure 2 shows a slightly abnormal E/Em ratio (= 9) in a transplanted patient (normal cut-off value < 8) [19].

#### Index of Myocardial Performance (IMP)

IMP is an index which combines LV systolic and diastolic parameters (= (IVRT + ICT/LVET, where IVRT = isovolumic relaxation time, ICT = Isovolumic contraction time and LVET = left ventricular ejection time) and gives information about LV global performance [20]. The rationale for using IMP in this setting is that, during AAR, LV diastolic dysfunction can be accompanied by subtle abnormalities of LV systolic performance and these are potentially reflected in changes of systolic time intervals. Accordingly, IMP has demonstrated to be sensitive to initial alterations of cardiac function in paediatric patients with AAR [21,22]. In 20 HT adult recipients with grade 3A cellular rejection, there was a mean increase of IMP by 98% (p < 0.0001) during the AAR episode and a decrease to its baseline values after treatment; in addition, the change in IMP was independent of both baseline EF and EF changes during AAR [23]. IMP could be, therefore, an useful non-invasive indicator, in order to assess the impact of therapy for amelioration of AAR. However, in another experience [24], the comparison of intra-recipient changes in Doppler intervals between rejection and non-rejection states demonstrated prolongation of IVRT and shortening of ICT during AAR, with no change in the IMP. It is possible that during AAR IVRT fall is counterbalanced by ICT prolongation and this may result in no significant change of IMP.

#### Left ventricular mass

After HT, it is evident an increase in wall thickness and LV mass, which can be detected and followed during time by serial echocardiographic examinations. LV hypertrophy is due to several causes (repetitive rejections, arterial hypertension, immunosuppressive therapy, chronic tachycardia and denervation) and its progression occurs mainly in relation to cyclosporine levels and blood pressure levels [25]. It is worthy of note that severe LV hypertrophy predicts mortality at 1-year follow-up in HT recipients [26].

#### Valvular heart disease

Valvular regurgitation is a fairly common occurrence immediately post HT but structural abnormalities of the aortic and mitral valves are not observed frequently after HT. In a long-term follow-up study, only 13 of 65 patients had mitral valve regurgitation (no patients had severe regurgitation), three cases of aortic valve regurgitation were reported and no patient has significant aortic valve stenosis [10]. Possible causes of mitral regurgitation include papillary muscle ischemia and the effect of multiple AAR episodes.

#### Tricuspid valve regurgitation

In contrast to the left-sided valves, tricuspid valve regurgitation is very common after HT, its etiology being multifactorial. When its appearance is early after HT, probably it is related to elevated pulmonary arterial pressures and vascular resistance in the recipient as well as to atrial structure and function (in patients who had undergone standard biatrial surgical approach). In the majority of the cases, the resolution of tricuspid regurgitation is possible within 1 month after HT, hand in hand with the normalisation of pulmonary arterial pressures [27]. Tricuspid regurgitation may be also due to injury of the chordal apparatus caused by the repeated EMBs. Biopsy-induced damage includes flail tricuspid leaflets and severe eccentric jets of regurgitations. A direct correlation between number of biopsies and severity of tricuspid regurgitation has been demonstrated: a number <31 EMBs significantly predicts a reduced risk of severe tricuspid regurgitation [28]. Regardless of cause, the persistence of tricuspid regurgitation is related to symptomatic right ventricular (RV) failure, impaired renal function and increased mortality [29].

#### The right ventricle

Because of the high prevalence of tricuspid valve regurgitation the assessment of RV systolic function is necessary in HT recipients. Although the majority of the cases of early RV dilation and associated hemodynamic change improve progressively in a matter of a week after HT [30], RV failure is a recognised cause of in-hospital death and some survivors have residual RV dilation. A relationship between the evidence of RV dysfunction and plasma B-type natriuretic peptide has also been observed [31]. In addition, even when RV systolic function appears normal, more subtle RV dysfunction can be unmasked by RV pulsed Tissue Doppler. In paediatric HT recipients, S_m _velocity of the lateral tricuspid annulus has demonstrated to be lower than in controls, showing a trend to a further reduction during time [32]. In the experience of Suderaswaran et al, similar to LV filling pressure, mean right atrial pressure was related weakly to routine tricuspid inflow variables but strongly to tricuspid E/E_m _[r = 0.79; n = 38; right atrial pressure = 1.76(E/E_m_) - 3.7] after HT (18) (Figure 3). In addition, in 18 of these patients who repeated right-sided cardiac catheterizations during time, the changes in mean right atrial pressure were well detected by Doppler, with a mean difference of 0 ± 3.45 mm Hg (18). In this view, pulsed Tissue Doppler of the tricuspid annulus should be recommended in the follow-up of transplanted patients [33].

#### Pericardial effusion

Moderate to large pericardial effusions, due to a mismatch between recipient and donor hearts or to the presence of AAR or even to the effect of some immunosuppressive drugs, occurs frequently during the early phase after HT. However, the sensitivity (= 49%) and specificity (= 74%) for diagnosis of AAR are limited [34] The possibility of an infective cause for a pericardial effusion should always been taken into account in these patients who are often immuno-depressed. Close echocardiographic monitoring is required in order to avoid heart tamponade. Pericardial effusion is not associated, however, with any adverse clinical outcomes and progressively disappears. If a large pericardial effusion accumulates slowly it may be of a little hemodynamic impact [35,36].

### Potential role of high-technology ultrasound tools

**Integrated backscatter (IBS) **appears able to identify AAR by the decrease of cyclic variation signal [37] and the increase of 2-D derived end-diastolic IBS signal of either posterior or septal wall [38]. The off-line analysis of colour Tissue Doppler and **strain rate imaging (SRI)**, a tool which quantifies myocardial wall deformation and distinguishes "true" active myocardial contraction from passive wall motion [39], has been experimented in HT recipient. "Healthy" HT patients may have normal global systolic function but altered regional systolic deformation compared to normal hearts [40]. SRI might be, therefore, an additional tool for detecting = IB grade of AAR [41] and reduce the number of EMBs. Despite promising, however, high-tech ultrasound technologies have been applied only on small number of HT recipients and their confirmation in larger population samples is needed.

### Acute allograft rejection

Several attempts have been performed during time to take advantage by M-mode and 2-D echocardiography to diagnose AAR. The main echocardiographic variables proposed for diagnosis of AAR include increased wall thickness and wall echogenity, pericardial effusion, LV diastolic dysfunction and regional/global LV systolic dysfunction [42]. Table 2 sumarises the sensitivity and specificity of Doppler echocardiography in the main studies performed [23,38,43-46]. In general, the results are not encouraging and, when an echocardiographic parameter appears appropriate, confirmatory results are lacking. Although in the absence of key specific echo abnormalities the probability of AAR is relatively low, no single echocardiographic variable alone may be used for accurate detection of AAR. Of interest, an experience of Picano et al [47] showed that histologically verified rejection is accompanied by normal global wall motion during stress-induced ECG ST-segment depression. This recalls the well known stress-echo response pattern of microvascular angina, characterized by normal epicardial coronary arteries and reduced coronary flow reserve (CFR), which can, indeed, acutely and transiently reduced by AAR [48]. Despite these evidences, Doppler echocardiography is not routinely used to diagnose AAR. It is expectable that the effect of bicaval procedure on Doppler, the development of new high-tech ultrasound technique and the use of transthoracic CFR could produce some effect of improvement in the ultrasound diagnostic accuracy of AAR in the future time.

**Table 2:** Main studies evaluating the accuracy of standard Doppler echocardiography in detecting AAR

**Authors**	**N. of patients**	**Method/Parameter**	**Sensitivity**	**Specificity**
Desruennes M, J Am Coll Cardiol 1988	55	Standard Doppler, PHT decrease (20%)	88%	87%
Simmonds MB, Circulation 1992	30	Standard Doppler, superior vena caval SFFV ≤ 17 cm/s	100%	80%
Morocutti G, J Heart Lung Transplant 1995	18	Standard Doppler, PHT ≤ 55 ms PHT ≤ 60 ms	69% 62%	76% 83%
Mouly-Bandini A, Transpl Int 1996	23	Standard Doppler, IVRT decrease ≥ 20%	45%	-
Angermann GE, Circulation 1997	52	M-mode and 2D parameters	40–55%	84–87%
Stengel SM, Heart 2001	141	Pulsed Tissue Doppler. A_m _of mitral annulus < 8.7 cm/s	82%	53%
Vivekananthan K, Am J Cardiol 2002	20	Standard Doppler, MPI	90%	90%
Sun JP, J Heart Lung Transplant 2005	223 183	Standard Doppler echo ≥ 2 among PE, IVRT > 90 ms, E/A > 1.7 post HT ≤ 6 months post HT > 6 months	57% 60%	54% 93%

IVRT = isovolumic relaxation time, MPI = Myocardial performance index, PE = pericardial effusion, PHT = Mitral pressure half-time, SFFV = inferior vena caval systolic forward flow velocity.

### Cardiac allograft vasculopathy

**Figure 4:**
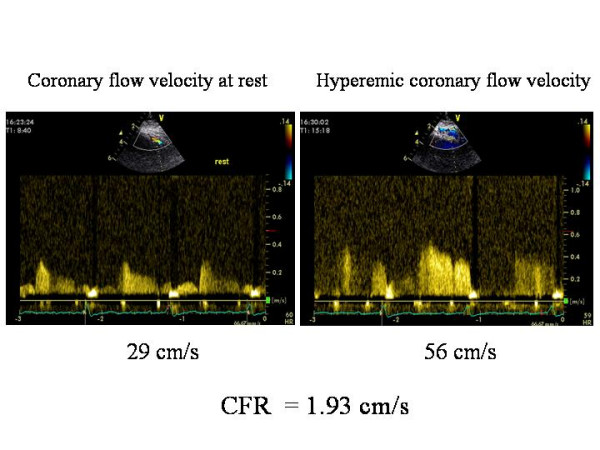
Reduced coronary flow reserve in a patient with coronary evidence of allograft vasculopathy.

The development of progressive CAD in HT has been recognised increasingly as long-term recipient survival has improved. Because CAV remains the major cause of death during long-term follow-up, its diagnosis is very important. It is remained for years in the prevalent domain of invasive techniques. Serial coronary angiography permits the study of coronary arteries and also the visualisation of coronary arteries by intravascular ultrasound (IVUS), that represents the reference gold standard in this clinical setting. Rapidly progressive vasculopathy by IVUS, defined as an increase of ≥ 0.5 mm in intimal thickness within the first year after HT, is a powerful predictor of all-cause mortality, myocardial infarction and angiographic abnormalities [47]. However, an annual angiographic evaluation is difficult to perform and, with almost equal reliability when compared with IVUS, CAV can be identified combining information on donor age, 2-D echocardiographically determined wall motion score at rest and immuno-fluorescence staining against anti-thrombin III in EMBs late after HT [49]. In this view, coronary angiography should be limited to patients with a high probability score and not be used routinely for surveillance of CAV. Clinical manifestations of CAV are often silent because the lack of afferent sympathetic innervation of the transplanted heart. Moreover, exercise performance in HT recipients has a limited value since the donor heart is surgically denervated without afferent parasympathetic or sympathetic innervation. Abnormalities of the ventricular rate response include resting tachycardia (due to parasympathetic denervation), a slow heart rate during mild-moderate exercise, a more rapid response during more strenuous exercise, and a more prolonged time for the ventricular rate to return to baseline during recovery. The heart rate response during exercise and the diffuse nature of CAV coronary abnormalities limit the sensitivity of ECG effort tests to detect coronary artery vasculopathy. Pharmacological stress echocardiography, a tool prognostically validated in groups of patients at risk for coronary artery disease [50], is safe and well tolerated in HT recipients. A varieties of stressors (adrenergic agents, vasodilators, cold pressure test) are available but dobutamine is preferred [51-58], mainly because denervation of the transplanted heart increases the responsiveness to chronotropic stimulation [59]. **Dobutamine stress echocardiography (DSE) **identifies patients at risk for events and facilitates monitoring of CAV. A normal DSE predicts an uneventful clinical course and justifies postponement of invasive studies [53,54,56]. Its prognostic value is comparable to that of IVUS and angiography [57]. DSE has been successfully proposed in order to predict cardiac events in long-term follow-up (4 years) of HT recipients [58]. The use of atropine after dobutamine infusion is controversial since it is evident that the heart of recipients is completely denervated and there are conflicting evidence regarding parasympathetic re-innervation. However, it is recent the demonstration that the adjunctive use of atropine in HT patients during stress-echo aids in reaching 85% of maximum predicted heart rate [60]. Also dipyridamole stress echocardiography is able to identify patients with altered wall motion who need careful surveillance and probably an invasive assessment [61,62]. Although wall motion at rest and after dipyridamole administration and CAV are predictors for cardiac events, only a wall motion score index >1 after dipyridamole remains significant at multivariate analysis [63]. Adenosine has been used to evaluate TTE-derived coronary flow reserve of left anterior descending artery (CFR) in transplant recipient, it showing 82% sensitivity, 87% specificity and 85% accuracy for CAV detection (cut-off point value ≤ 2.7) [64]. A shorter diastolic flow velocity at rest (deceleration time of diastolic velocity of ≤ 840 ms: sensitivity = 86%, specificity = 75%, positive predictive value = 33%, negative predictive value = 97%, p = 0.002) and a reduction of CFR (cut-off point of CFR ≤ 2.6, sensitivity = 91%, specificity = 62%, positive predictive value = 32%, negative predictive value = 97%, p = 0.001) have demonstrated to be both reliable markers for CAV-related major cardiac adverse events [65]. Figure 4 shows an abnormal CFR in a HT recipient. Of interest, dipyridamole-derived CFR is related positively with Tissue Doppler derived S_m _velocity and negatively with E/E_m _ratio in HT recipients: this findings indicates a possible association of impaired coronary microcirculation with both myocardial systolic dysfunction and increase of LV filling pressures in this clinical setting [66].

Table 2 sumarises the main studies reporting sensitivity and specificity of pharmacological stress in detecting CAV and coronary artery stenosis > 50% in HT recipients (26). In general, stress echocardiography appears as a very important tool to identify CAV in HT recipients.

### Echocardiography during endomyocardial byopsies

**Figure 5:**
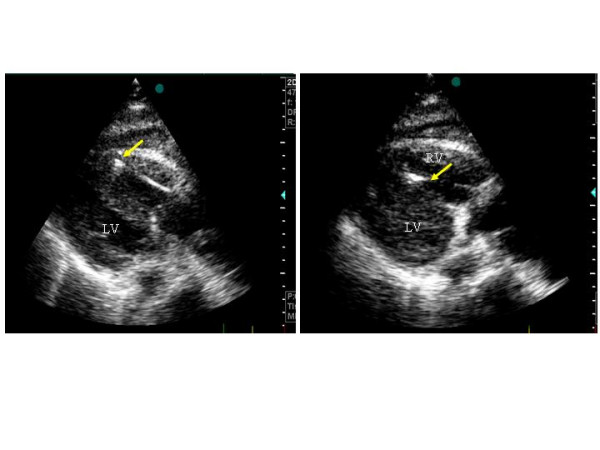
Use of 2-D echocardiography for monitoring the performance of endomyocardial biopsy in a HT recipient. The arrow indicates the site of the biopsy. Left panel: at the apex of right ventricle, right panel: al the level of the right side of the interventricular septum.

Another use of transthoracic echocardiography is the monitorisation and the visualisation of the catheter during the performance of endomyocardial biopsy [67]. The use of the ecocardiography is important to avoid an useless and dangerous exposition to X-ray and permits to follow adequately the movement of the catheter in the right ventricle and to select the site for biopsy (Figure 5). Echocardiography gives also the possibility to avoid damaging of tricuspid valve, papillary muscles or chords and to promptly identify the eventual presence of other complications like pericardial effusion. Real time 3-D echocardiography seems very promising in improving the ability to see the location of the bioptome during EMBs compared with 2-D echocardiography and fluoroscopy [68,69].

### Heart donor storage

Heart donor storage is a main problem since patients in HT waiting list have a 7.3% death rate and the average waiting time is 2 to 3 years. In addition, there is a large amount of ''marginal'' recipients, it being due to either advanced age (> 65 years) or co-morbidity. Of consequence, a gradual trend toward liberalizing donor selection criteria has been developed and an expansion of the cardiac donor pool has involved accepting hearts of older donors, tolerating longer organ ischemic times and accepting hearts with structural and/or functional abnormalities, such as mild LV hypertrophy and mild valvular abnormalities [70-72]. In this view, although it is fair to recognize that transthoracic ultrasound imaging can be suboptimal in several patients on ventilators, the role of echocardiography has became crucial in order to detect adequate LV function and lack of significant valvular heart disease in the potential donors. Donors with echocardiographic functional abnormalities at the time of donation present an excellent 4 years survival rates [73]. The most important problem for an adequate selection is, however, the need of excluding donors with more than mild coronary artery disease. Coronary angiography is recommended for the majority of male donors older than 45 years and female donors older than 50 years, in order to exclude significant coronary artery stenosis. A simpler approach should be represented by bedside, pharmacological stress echocardiography, whose feasibility has been recently validated in potential heart donors with brain death: an aged donor with normal resting and stress echo, without regional wall motion abnormalities or with an hyperkinetic global response to the stress, can be judged as a good candidate for an uneventful HT [74]. The identification of hearts ''too good to die'' on the basis of bedside resting and stress echo can be a critical way to solve the mismatch between donor need and supply. Comparison between pre-transplant donor stress-echo and post-transplant recipient stress-echo could be performed to assess normal or abnormal function of the graft.

**Table 3:** Main studies evaluating the accuracy of pharmacological stress echocardiography in detecting CAV and coronary artery stenosis > 50% in HT recipients (Mod from Thorn EM et al, *Heart Fail Clin *2007)

**Authors**	**N. of pts**	**Time post-HT (years)**	**Sensitivity for CV**	**Specificity for CAV**	**Sensitivity for cor. stenosis**	**Sensitivity for cor. stenosis**
**Dobutamine**						

Akosah KO, 1994	41	4.8 (0.25–10)	95%	50%	100%	41%
Herregods, J Heart Lung Tr1994	28	3.2 ± 1.3	50%	71%	-	-
Derumeaux G, JACC 1995	41	3.3 ± 1.7	86%	91%	100%	77%
Derumeaux G, Arch Mal Coeur 1996	64	3.3 ± 1.2	85%	97%	100%	-
Akosah KO, JACC 1998	22	0.17 (0.04–0.3)	100%	73%	100%	59%
Derumeaux G, J Heart Lung Tr 1998	37	3.1 ± 1.7 4.7 ± 1.8	65% 92%	95% 73%	- -	- -
Spes GH, Circulation 1999	109	3.2 ± 3.1	72%	88%	-	-
Bacal F, J Heart Lung Tr 2004	38	> 4	-	-	64%	91%

**Dipyridamole**						

Ciliberto GR, Eur Heart J 1993	80	2.3 ± 0.5	80%	85%	100%	72%
Ciliberto GR. J Heart Lung Tr 2003	68	2.9 ± 1.9	80%	79%	100%	87%

**Adenosine**						

Tona F J Heart Lung Tr 2006	73	8.0 ± 4.5	82%	87%	-	-

CFR = Coronary flow reserve

## Conclusion

Transthoracic Doppler echocardiography is a primary non-invasive modality for investigation of cardiac transplant recipients. It is a versatile tool which provides comprehensive information about cardiac structure and function. Echocardiographic examinations can be easily performed at the bedside and serially repeated without any patient's discomfort. Although sustained efforts to develop and echocardiographic technique able to predict the biopsy state have been performed, it has fair to recognise that EMBs are still regarded as the gold standard for detection of acute allograft rejection. Conversely, stress echocardiography is able to identify accurately CAV and has recognised prognostic value, which is comparable to that of IVUS or angiography. A normal stress-echo justifies postponement of invasive studies. Bedside stress echocardiography is even useful to select appropriately heart donors with brain death. Finally, echocardiographic monitoring is simple and effective for monitoring a safe performance of biopsy procedures.

## Competing interests

The author(s) declare that they have no competing interests.

## Authors' contributions

SM conceived the study, participated in its design and drafted the manuscript, MM and MG reviewed the manuscript and participated in the design of the study, All the authors read and approved the final manuscript.
